# Post-allogeneic stem cell transplant *FLT3*- targeted maintenance therapy: updates and considerations for clinical practice

**DOI:** 10.46439/stemcell.3.015

**Published:** 2022

**Authors:** Jonathan Cohen, Richard T. Maziarz

**Affiliations:** 1 Knight Cancer Institute, Oregon Health & Science University, Portland, OR USA; 2 Department of Pharmacy, Oregon Health & Science University, Portland, OR USA; 3 Center for Hematologic Malignancies, Oregon Health & Science University, Portland, OR USA

**Keywords:** Allogeneic stem cell transplant, FLT3, Maintenance

## Abstract

Acute myeloid leukemia (AML) is characterized by multiple molecular and cytogenetic abnormalities, with increasing data to support clinical and prognostic implications to guide clinical decision making. One of the most well described mutations involves fms-like tyrosine kinase 3 (*FLT3*) that results in a constitutively active tyrosine kinase and is generally associated with poor prognosis involving shorter overall survival and higher rates of relapse. Advancements in targeted therapies have greatly influenced available treatment options in a landscape that has remained largely unchanged for the past five decades. Tyrosine kinase inhibitors (TKI), specifically *FLT3*-targeted therapies, are now integral treatment options for patients with this targetable mutation. As allogeneic hematopoietic cell transplant (alloHCT) remains the primary curative therapy for most adult AML patients, the goal is for eligible patients to proceed to transplant. However, post-alloHCT relapse remains exceedingly high even in patients achieving deep responses to therapy. Limited evaluation of *FLT3*-targeted TKIs as post-alloHCT maintenance therapy in *FLT3*-positive patients suggest improved outcomes and tolerable safety profiles, with ongoing studies further investigating second-generation agents. Thus, this commentary aims to review the role of post-alloHCT *FLT3*-targeted maintenance therapy and considerations for clinical practice.

## Introduction

Acute myeloid leukemia (AML) is estimated to account for about one-third of new leukemia cases in 2022 [1,2]. AML is characterized by multiple molecular and cytogenetic abnormalities, with data to support that these well-defined genomic mutations have prognostic importance [3]. As a consequence of these detailed characterizations of the AML genomic landscape, there has been an emergence of molecularly targeted therapies over the past decade. These innovations offer new therapy options for AML, amidst standards of care that have otherwise, remained largely unchanged over the past 50 years [4]. However, despite these recent advancements, outcomes remain poor with an estimated 5-year relative survival of ~25–30% [2].

Allogeneic hematopoietic cell transplant (alloHCT) remains the primary curative therapy for most adult AML patients, particularly in those individuals who achieve complete remission (CR), due in part to the immunologic benefit of the associated graft versus leukemia (GVL) effect. However, relapse remains exceedingly high in certain patient populations, such as in individuals with treatment-related AML, monosomal karyotype or complex cytogenetics on presentation, or those with detectable minimal residual disease (MRD) after induction/consolidation therapy. Currently, high relapse risk populations are also increasingly being identified upfront with next generation sequencing, targeting known myeloid gene panels. One well-defined mutation in AML involves the fms-like tyrosine kinase 3 (*FLT3*), occurring in 30% of adult AML patients [5]. Generally, *FLT3* AML has been associated with poor prognosis involving shorter overall survival and higher rates of relapse, due in part to its role as a driver mutation and contribution to possible clonal evolution [5–7]. As a result, subjects with *FLT3*^+^ AML are generally considered for alloHCT. Given the associated risk with *FLT3*-positivity, development of second-generation *FLT3*-targeted therapies, and understanding of the critical nature of MRD status, there is growing interest in continuation of *FLT3*-targeted therapies following alloHCT in an effort to deepen remission and prevent relapse.

## FLT3 Mutations in AML

Whole genome sequencing of multiple AML patient samples has revealed increasing numbers of genetic mutations. However, the *FLT3* gene remains one of the most frequently identified mutated genes [8–10]. *FLT3* mutations generally present with either (1) in-frame internal tandem duplication (ITD) or (2) point mutations within the tyrosine kinase domain (TKD) [5]. Either phenotype results in constitutive activity of the transmembrane tyrosine kinase, promoting aberrant cell growth and survival. Internal tandem duplication is more prevalent (25% of AML cases) and generally confers a more unfavorable prognosis compared to the less well defined *FLT3*-TKD (5–10% of cases) [5]. Furthermore, higher degree of *FLT3*-ITD allelic ratio confers increased risk with concomitant expression of other mutations, namely *NPM1* [4,5].

Comparative studies evaluating clonal evolution of patients with relapsed AML suggest that *FLT3*-ITD mutations may likely confer a selective survival advantage within the tumor microenvironment. One study reported persistent *FLT3* -ITD clones in 75% of patients at the time of relapse [5,11]. These clinical findings have supported the development of *FLT3*-targeted therapy with high specificity and binding affinity (discussed below).

## FLT3-Targeted Therapy

There are now several *FLT3*-targeted therapies available. Initial studies evaluated use of first generation multi-targeted kinases including sunitinib and sorafenib [5]. Both agents were evaluated in phase 1 or phase 2 studies with mixed results given limited anti-leukemic activity and poorer tolerability as single-agent therapy. Combination therapy with high-intensity chemotherapy also had limited application to older patient populations after one study found no improvement in survival and increased mortality due to infection [5]. However, current guideline recommendations support use of sorafenib in combination with a hypomethylating agent in patients who are *FLT3*-ITD positive and not candidates for transplant nor able to receive high-intensity therapy [12].

The major breakthrough involving *FLT3*-targeted therapy was the phase 3 RATIFY/CALGB 10603 trial of midostaurin, another tyrosine kinase inhibitor (TKI) that targets *FLT3*, in combination with conventional induction (7+3) chemotherapy for the treatment of newly diagnosed *FLT3*-mutated AML. Improved event-free survival (EFS; Hazard Ratio (HR) 0.78; P = 0.002) and overall survival (OS; HR 0.78; P = 0.009) was shown compared to conventional chemotherapy alone [5,13]. Many patients in the RATIFY trial proceeded to alloHCT, but notably these patients did not continue post-transplant midostaurin. However, despite the relative success of midostaurin, relapse remained significant. Thus, more potent, second-generation agents gilteritinib, crenolanib, and quizartinib have been developed and have shown promise in relapsed/refractory *FLT3*^+^ AML and appear to remain effective as single-agent therapy [5].

## FLT3-Targeted Maintenance Therapy Post-AlloHCT

Given the significant findings related to peri-transplant MRD positive or negative status and significant rates of relapse with *FLT3*^*+*^ AML, there has been significant interest in ongoing *FLT*-directed maintenance therapy after alloHCT while waiting to establish the donor GVL effect. Sorafenib was first evaluated in a phase I dose-escalation study by the Mass General Hospital group, in which patients received sorafenib 45–120 days post-alloHCT for up to 12 28-day cycles [14]. Overall, 22 patients were enrolled, of which 3 relapsed (2 with primary refractory AML prior to alloHCT; 1 in CR prior to alloHCT). The majority of patients (n=19) were in CR1/CR2 prior to alloHCT with a 1-year progression-free survival (PFS) and OS of 95% (90% CI, 76% to 99%) and 100%, respectively [14]. A follow-up study conducted by Brunner and colleagues sought to affirm the results of the phase I study. Overall, 26 patients received sorafenib (n=16 included from the phase I study) compared to 55 controls [15]. Of note, a landmark analysis was conducted to include only control patients alive at the median time of sorafenib initiation (n=43) to account for patients with early relapse. In the entire cohort with sorafenib exposure as a time-varying covariate, sorafenib patients experienced improved OS (HR 0.264; P=0.021) and PFS (HR 0.25; P=0.016). In the landmark analysis, sorafenib patients similarly had improved 2-year OS (81% vs 62%; P=0.029), 2-year PFS (82% vs 53%; P=0.0081) and decreased 2-year incidence of relapse (8.2% vs 37.7%; P=0.0077). Although 42% of sorafenib patients discontinued therapy prior to 12 months, there was no difference in 2-year non-treatment related mortality (NRM; 9.8% vs 9.3%; P=0.82) or chronic graft-versus host disease (cGvHD rates; 55.5% vs 37.2%; P=0.28). Since this time, other retrospective studies have similarly suggested survival benefit and lower incidence of relapse with use of maintenance sorafenib post-alloHCT [16–18].

Based on these initial observations, two randomized, controlled trials of maintenance sorafenib have been performed. The positive results have led to NCCN and EBMT endorsement of sorafenib use post-alloHCT in AML patients with history of *FLT3*-ITD in remission [12,19,20]. Specifically, the phase II SORMAIN trial was a randomized, double-blind, placebo-controlled study of post-alloHCT sorafenib maintenance starting D+60 to D+100 in patients with a history of *FLT3*-ITD with complete hematologic remission (bone marrow blasts <5%) [21]. Patients were randomized to receive a 24-month course of either sorafenib (400 mg twice daily, reflecting maximum tolerated dose from prior retrospective studies) or placebo. Overall, 83 patients were randomized (sorafenib n=43, placebo n=40). The study was closed early due to slow accrual; however, results remained encouraging with an estimated probability of 24-month RFS and 24-month OS of 85% versus 53% and 90.5% (95% CI, 77% to 96%) versus 66.2% (95% CI, 49% to 79%) for sorafenib versus placebo, respectively (HR of death 0.241; 95% CI 8% to 74%; log-rank P=0.007). A larger open-label phase III trial was conducted randomizing patients to either sorafenib or no maintenance therapy 30–60 days post-alloHCT in patients with hematopoietic recovery by D+60. Overall, 202 patients were randomized (sorafenib n=100, placebo n=102) [22]. Patients who received sorafenib had lower incidence of relapse compared to the control group 1-year post-alloHCT (7% (95% CI 3.1% to 13.1%) vs 24.5% (95% CI 16.6% to 33.2%); HR 0.25 (95% CI 0.11 to 0.57; P = 0.0010)). Sorafenib appeared to be better tolerated than previously reported in the phase II study, with similar rates of aGvHD and cGvHD between sorafenib and control groups (23% vs 21% and 18% vs 17%, respectively).

Although the majority of post-alloHCT *FLT3*-targeted maintenance therapy describes sorafenib, there are emerging data that support use of midostaurin. The phase II AMLSG 16–10 trial evaluated use of midostaurin in combination with intensive chemotherapy similar to the RATIFY trial, but permitted continuation of single-agent midostaurin as maintenance therapy starting as soon as 30 days post-alloHCT [23]. Of the 284 patients who received induction therapy, 75 received post-alloHCT maintenance therapy. Compared to non-transplant patients (patients in CR who received high-dose cytarabine) receiving maintenance therapy, alloHCT recipients experienced less relapse at 2 years (13.3% vs 43.5%, respectively). Additionally, alloHCT patients who started maintenance therapy prior to D+100, were event-free at D+100, and in CR1/CRi prior to alloHCT had significantly better EFS (P=0.01, univariable; P=0.004, multivariable) and OS (P=0.02, univariable; P=0.01, multivariable) compared to patients who started maintenance therapy after D+100. Findings from AMLSG 16–10 affirmed the importance of MRD status peri-alloHCT and suggested that early initiation (prior to D+100) of maintenance therapy should be implemented, if possible. More recently, the phase II RADIUS trial was the first randomized control trial conducted to assess post-alloHCT maintenance midostaurin [24]. RADIUS was an open-label trial that randomized patients to either no maintenance or midostaurin starting D+28 to D+60 for 12 4-week cycles. The results demonstrated a trend to improved RFS at 18 months (HR 0.46 (95% CI 0.12- to 1.86), P =0.27) and OS at 24 months (HR 0.58 (95% CI 0.19 to 1.79), P=0.3418), with estimated RFS and OS of 89% (95% CI 69% to 96%) versus 76% (95% CI 54% to 88%) and 85% versus 76% for midostaurin and the control group, respectively. The authors note that a statistically significant improved RFS was observed in the 50% of patients who received midostaurin that achieved inhibition of *FLT3* phosphorylation to <70% of baseline. The inability to achieve adequate inhibition through the entire patient cohort may have been impacted from adverse effects related to midostaurin, with 63% of patients requiring dose adjustments and 8 patients requiring discontinuation of therapy. Also, it was noted that the control group (76% OS) had better outcomes than otherwise seen historically or in other *FLT3* post-alloHCT studies, potentially influencing the outcomes seen.

A recent systematic review and meta-analysis assessing post-alloHCT *FLT3*-targeted therapies supported improved outcomes with generally well-tolerated adverse effects and no overt safety concerns related to post-transplant complications [25]. The analysis included 680 patients from 7 studies (5 sorafenib and 2 midostaurin). Relapse was significantly improved with use of *FLT3* maintenance therapy compared to placebo (pooled risk ratio (RR) = 0.35 (95% CI 0.23 to 0.51), P<0.001). Pooled RR for RFS (RR=0.48 (95% CI 0.37 to 0.61), P<0.001) and OS (RR=0.48 (95% CI 0.36 to 0.64), P<0.001) were also significantly improved. There was no difference in NRM or GvHD. Similarly, a large-scale retrospective review across 8 countries involving 1208 patients identified 219 patients who received *FLT3*-targeted maintenance therapy [26]. Maintenance therapy observed improved RFS (adjusted HR 0.57 (95% CI 0.34 to 0.94), P<0.05) and OS (adjusted HR 0.50 (95% CI 0.28 to 0.89), P<0.05).

Second generation *FLT3* inhibitors with greater specificity may be ideal for post-alloHCT maintenance therapy. Currently, only gilteritinib is approved in the United States for use in relapsed/refractory disease and is currently under investigation for use as maintenance therapy in the phase III BMT CTN 1506 trial (NCT02997202), with analysis pending [27]. There are significant differences in coordination of this research trial as opposed to the prior studies (e.g. SORMAIN, RADIUS). With the FDA label for midostaurin as part of induction and consolidation therapy for *FLT3*-ITD^+^ AML patients, most subjects will have already been exposed to a TKI. This specific post-alloHCT trial randomizes patients, after engraftment, to placebo versus gilteritinib. Maintenance continues for 2 years after alloHCT. The primary objective is to compare RFS between the gilteritinib and the placebo arms with secondary objectives of tolerability, OS, aGVHD and exploratory objectives of quality of life analysis, healthcare resource utilization, study drug pharmacokinetics, and determination of *FLT3* status at relapse.

Other studies examining second generation *FLT3* inhibitors are smaller in scope. A phase I dose escalation study of quizartinib included 13 post-alloHCT patients with planned maintenance therapy of up to 24 months [28]. One patient relapsed, with 5 patients completing the full 24 months of therapy and 4 patients discontinuing therapy due to adverse effects. Subset analysis of the phase III QuaANTUM-R study of quizartinib in relapse/refractory AML suggests that patients who receive quizartinib may be more likely to proceed to alloHCT. Patients who received alloHCT had improved median OS compared to those who did not (12.2 months vs 4.4 months, respectively; HR 0.315 (95% CI 0.233 to 0.427)) [29]. Patients with composite CR prior to alloHCT also had improved survival (20.1 months vs 8.8 months; HR 0.5066 (95% CI 0.296 to 0.864)). A proportion of patients continued post-alloHCT quizartinib and did not appear to have any overt safety or tolerability concerns. Finally, there is limited experience with crenolanib, with ongoing trials assessing use as maintenance therapy (NCT02400255, NCT03258931).

## Future Considerations

There remain several ongoing clinical trials seeking to further investigate use of *FLT3*-targeted therapy as post-alloHCT maintenance therapy. Encouraging results thus far suggest improved survival and lower incidence of relapse with targeted maintenance therapy after alloHCT. The data obtained consistently demonstrate that patients with CR prior to alloHCT with MRD^−^ status appear to have better outcomes. Early use of alloHCT maintenance therapy is supported, recognizing that relapse often occurs early (<100 days) [24]. With the observed apparent benefits of maintenance therapy and high risk of relapse in *FLT3*-ITD patients, some ethical concerns may exist related to continued investigation involving placebo, as carefully outlined by Levis et al [30]. Yet, with better tolerability of the second generation TKIs used as maintenance therapy, less patients may discontinue therapy or require dose reductions due to adverse effects. Thus, it is important to weigh the risks and benefits of continued maintenance therapy.

Finally, we must recognize that there are cost considerations which justify the needs for ongoing clinical trials. AlloHCT is an expensive procedure. One recent analysis of a payor claims data base revealed a median of ~$417,000 adjudicated claims payments for the first year of coverage [31]. The Milliman group reported charges of ~ $930,000 from 30 days prior to alloHCT through day 180 [32]. Thus, it will be important to acknowledge the additional cost of TKI administration, recognizing that in the trials that were discussed herein, that the maintenance treatment extended for up to 2 years. These costs will be balanced against the cost of relapse care (reinduction therapy, donor leukocyte infusion vs second transplant) [33]. Specifically, recent available data re: Average Wholesale Price (AWP) of the TKIs are worthy of examination: [34].

**Table:** 

Drug	Formulation/Unit	Cost per Unit (AWP)	Estimated Annual Cost^†^
Midostaurin	25 mg capsule	$213.90	$287,481.60
Sorafenib	200 mg tablet	$221.93	$298,273.92
Gilteritinib	40 mg tablet	$343.59	$346,338.72

AWP: Average Wholesale Price;

† Annual cost estimated with full dose of specified drug (e.g. midostaurin 50 mg twice daily, sorafenib 400 mg twice daily, or gilteritinib 120 mg daily) over 12 28-day cycles.

Health resource utilization analysis re: the cost of care is needed to best understand what are the true optimal outcomes, analyses often extending beyond RFS to quality-adjusted life years (QALY) gained by an intervention (focusing on length of survival and health-related quality of life) and the increasingly recognized, critical need for patient reported outcome (PRO) measures.

## Conclusion

Routine use of *FLT3*-targeted post-alloHCT maintenance therapy continues to be an area of interest and ongoing investigations. Guideline recommendations support use of sorafenib as maintenance therapy in patients with a history of *FLT3*^*+*^ disease. Encouraging results suggest similar application of midostaurin, with analysis of second-generation therapies expected to mature in the near future. Patients in CR prior to alloHCT may be ideal candidates to start early maintenance therapy in an effort to maintain MRD negativity, but it is important to note that use of maintenance therapy is not benign given potentially significant adverse effects and costs to the health care system.
